# A Conformational Change in the N Terminus of SLC38A9 Signals mTORC1 Activation

**DOI:** 10.1016/j.str.2020.11.014

**Published:** 2020-12-08

**Authors:** Hsiang-Ting Lei, Xuelang Mu, Johan Hattne, Tamir Gonen

**Affiliations:** 1 Howard Hughes Medical Institute, University of California, Los Angeles, Los Angeles, CA 90095, USA; 2 Molecular Biology Institute, University of California, Los Angeles, Los Angeles, CA 90095, USA; 3 Departments of Biological Chemistry and Physiology, David Geffen School of Medicine, University of California, Los Angeles, Los Angeles, CA 90095, USA; 4 These authors contributed equally; 5 Lead Contact

## Abstract

mTORC1 is a central hub that integrates environmental cues, such as cellular stresses and nutrient availability to modulate metabolism and cellular responses. Recently, SLC38A9, a lysosomal amino acid transporter, emerged as a sensor for luminal arginine and as an activator of mTORC1. The amino acid-mediated activation of mTORC1 is regulated by the N-terminal domain of SLC38A9. Here, we determined the crystal structure of zebrafish SLC38A9 (drSLC38A9) and found the N-terminal fragment inserted deep within the transporter, bound in the substrate-binding pocket where normally arginine would bind. This represents a significant conformational change of the N-terminal domain (N-plug) when compared with our recent arginine-bound structure of drSLC38A9. We propose a ball-and-chain model for mTORC1 activation, where N-plug insertion and Rag GTPase binding with SLC38A9 is regulated by luminal arginine levels. This work provides important insights into nutrient sensing by SLC38A9 to activate the mTORC1 pathways in response to dietary amino acids.

## In Brief

SLC38A9 is an amino acid sensor in mTORC1 signaling through a process that is largely unknown. In this study, Lei et al. describe the structure of SLC38A9 with its N terminus plugged and propose a ball-and-chain mechanism of mTORC1 activation and arginine-enhanced transport by SLC38A9.

## Graphical Abstract

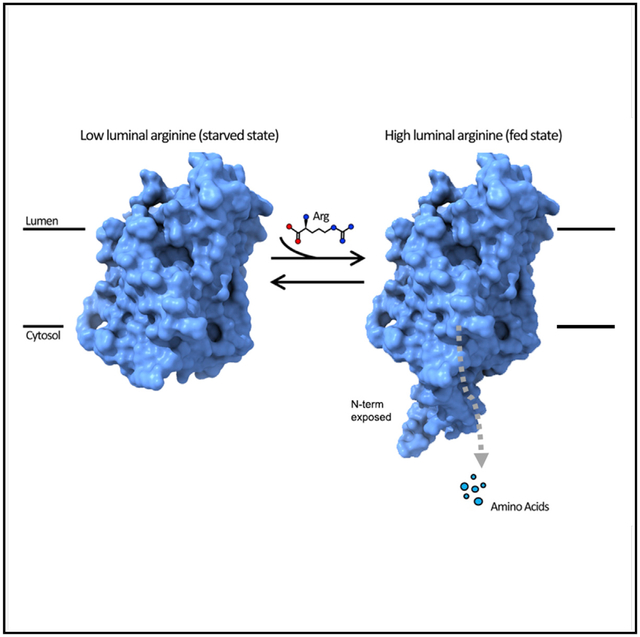

## INTRODUCTION

The mechanistic target of rapamycin complex 1 (mTORC1) protein kinase acts as a central signaling hub to control cell growth and balance the products from anabolism and catabolism (Ben-Sahra and Manning, 2017; Saxton and Sabatini, 2017; Shimobayashi and Hall, 2014). Not surprisingly, this pathway is dysregulated in many diseases (Laplante and Sabatini, 2012; Zoncu et al., 2011b). Activation of mTORC1 is mediated by a variety of environmental cues, such as nutrient availability, cellular stresses, and energy levels (Dibble and Manning, 2013; Sengupta et al., 2010). Specifically, certain amino acids signal to mTORC1 through two Ras-related guanosine triphosphatases (GTPases) (Kim et al., 2008; Sancak et al., 2008). When amino acids are abundant, the heterodimeric Rag GTPases adopt an active state and promote the recruitment of mTORC1 to the lysosomal surface (Sancak et al., 2010), which is now recognized as a key subcellular organelle involved in mTORC1 regulation (Zoncu et al., 2011a). Several essential amino acids in the lysosomal lumen, including arginine, leucine, and glutamine have been identified as effective activators of mTORC1 (Goberdhan et al., 2016; Jewell et al., 2013; Shimobayashi and Hall, 2016; Wolfson and Sabatini, 2017). However, the molecular basis of the amino acids-sensing mechanism has remained, by and large, elusive. Recently, SLC38A9, a low-affinity arginine transporter on lysosome vesicles, was identified as a direct sensor of luminal arginine levels for the mTORC1 pathway (Jung et al., 2015; Rebsamen et al., 2015; Wang et al., 2015). SLC38A9 also mediates the efflux of essential amino acids from lysosomes, such as leucine, in an arginine-regulated manner (Wyant et al., 2017), to drive cell growth by modulating cytosolic sensors (Saxton et al., 2016; Wolfson et al., 2016). Moreover, SLC38A9 senses the presence of luminal cholesterol and activates mTORC1 independently of its arginine transport function (Castellano et al., 2017).

SLC38A9 is a transceptor. Studies showed that two parts of SLC38A9, its N-terminal domain and its transmembrane (TM) bundle, are responsible for two distinct functions. The bulk of SLC38A9 are 11 α helices that pack against one another forming a TM bundle that transports amino acids and functions as an amino acid transporter (Lei et al., 2018). The N terminus of SLC38A9, on the other hand, was previously shown to interact directly with the Rag-Regulator complex to activate mTORC1 (Wang et al., 2015). Collectively, these results suggest that SLC38A9 is a “transceptor,” which is a membrane protein that embodies the functions of both a transporter and a receptor (van den Berg et al., 2016; Hundal and Taylor, 2009; Lei et al., 2018; Popova et al., 2010; Van Zeebroeck et al., 2009). Signaling, however, may or may not involve substrate transport.

We recently solved the crystal structure of N-terminally truncated SLC38A9 from *Danio rerio* (ΔN-drSLC38A9) with arginine bound (Lei et al., 2018). The substrate arginine was observed deep in the transporter at a binding pocket consisting of residues from TM1a, TM3, and TM8 of SLC38A9. Because the N-terminally truncated form of SLC38A9 was used, the initial study focused solely on the transporter function of SLC38A9 and the resulting structures could not inform on the signaling function of SLC38A9. Here, we report a crystal structure of drSLC38A9 with its N terminus but without the substrate arginine. Surprisingly, we found that a section of the N terminus formed a β hairpin that lodged itself deep within the transporter occupying the arginine binding site. These results suggest that, in the presence of high luminal arginine levels, the N-terminal domain could be displaced from the binding pocket by arginine and freed to interact with the Rag GTPase to activate mTORC1. We propose a ball-and-chain model to describe this mechanism of amino acid sensation and signaling by SLC38A9.

## RESULTS

**Figure 1.**
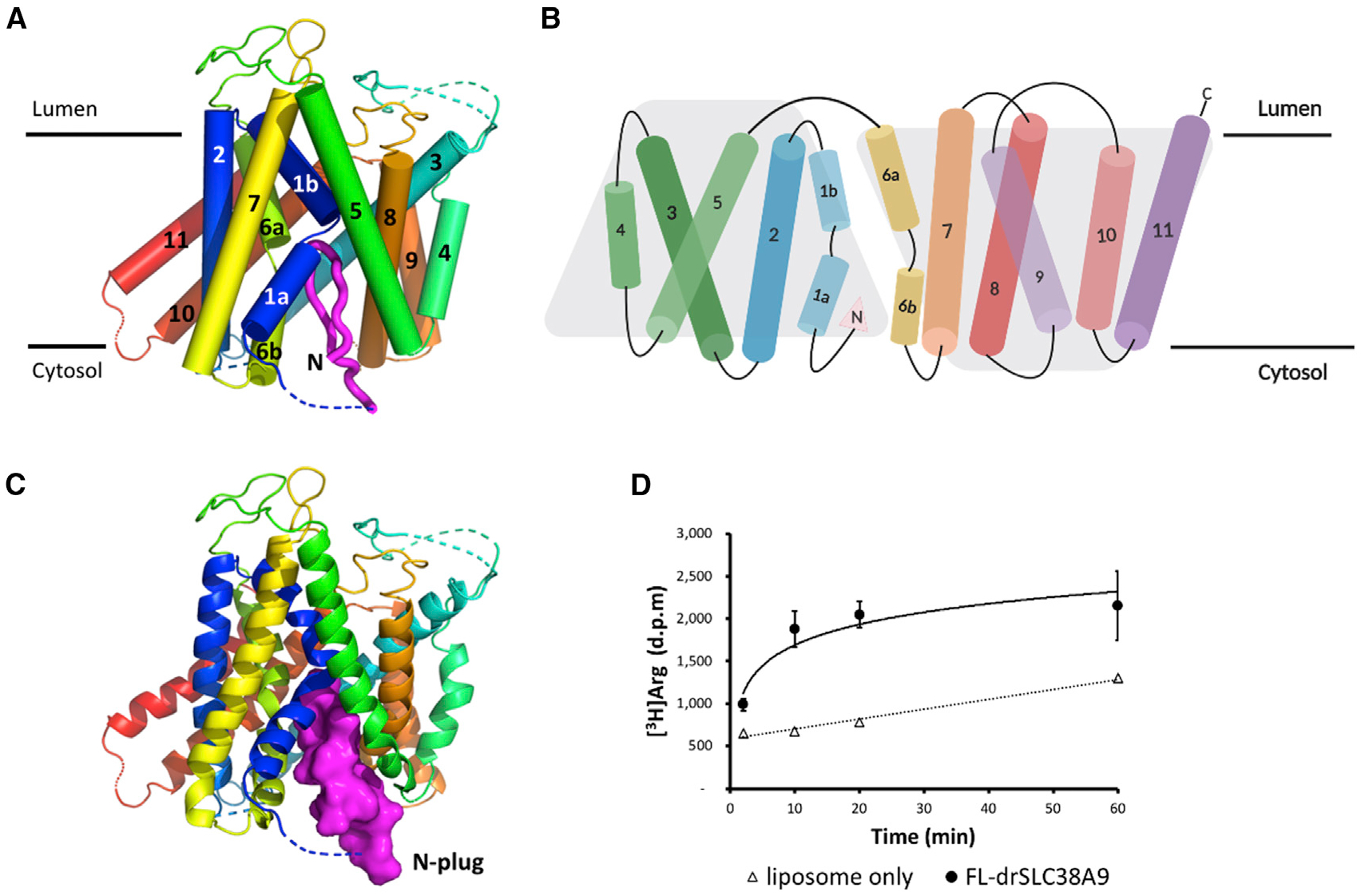
Structure of drSLC38A9 in the N-Plug Inserted State (A) View in the plane of the membrane. TMs are rainbow-colored as blue to red from N to C terminus. The N-plug is shown in magenta. (B) Two-dimensional topology model of drSLC38A9, which is folded into a characteristic 2-fold LeuT-like pseudo-symmetry (five transmembrane-helix inverted topology repeat). N-plug is marked by a filled pink triangle, next to the TM1a helix. (C) The N-plug blocks an otherwise cytosol-open state of drSLC38A9. (D) Time course of [^3^H]arginine uptake at 0.5 μM by proteoliposomes reconstituted with purified full-length drSLC38A9. Error bars represent standard error of the mean (SEM) of triplicate experiments.

We used the antibody fragment 11D3 to facilitate the crystallization of drSLC38A9 in the absence of substrate. Well-ordered crystals were diffracted to ~3.4Å with high completeness and acceptable refinement statistics (Table 1). Each asymmetric unit contained two copies of the drSLC38A9-Fab complex, arranged in a propeller-like head-to-head fashion (Figure S1). As with the recently determined structure (Lei et al., 2018), the TM domain of drSLC38A9 was captured in the cytosol-open state and was folded into the same inverted topology repeats made up of TMs 1–5 and TMs 6–10, with TM11 wrapping around the transceptor (Figures 1A and 1B). The two structures shared an overall similar fold with a root-mean-square deviation of 0.8 Å. However, instead of an arginine molecule bound, this time an unexpected electron density was observed, which extended along the solvent-accessible tunnel leading from the substrate-binding site to the cytosolic side of drSLC38A9 (Figure S2). The density was of sufficient quality to allow an unambiguous assignment of the drSLC38A9 N-terminal section from Asp75 to Leu90 (Figure S3). This fragment formed a folded domain, resembling a β hairpin, filling the entire path from the cytosolic side of SLC38A9 to the substrate-binding site (Figure 1C).

The binding of the N-terminal domain (referred to as the “N-plug” from this point on) inside drSLC38A9 does not appear to be a crystallization artifact. No crystal contacts exist near the N-plug, and the crystal has 40% solvent content and has not undergone dehydration, suggesting that the N-plug motif is not artificially displacing water within the crystal. Electrostatic potential analysis indicated that the transport pathway in drSLC38A9 is generally positively charged, while the N-plug is largely negatively charged (Figure S4), suggesting that the interaction is electrostatically driven.

**Figure 2.**
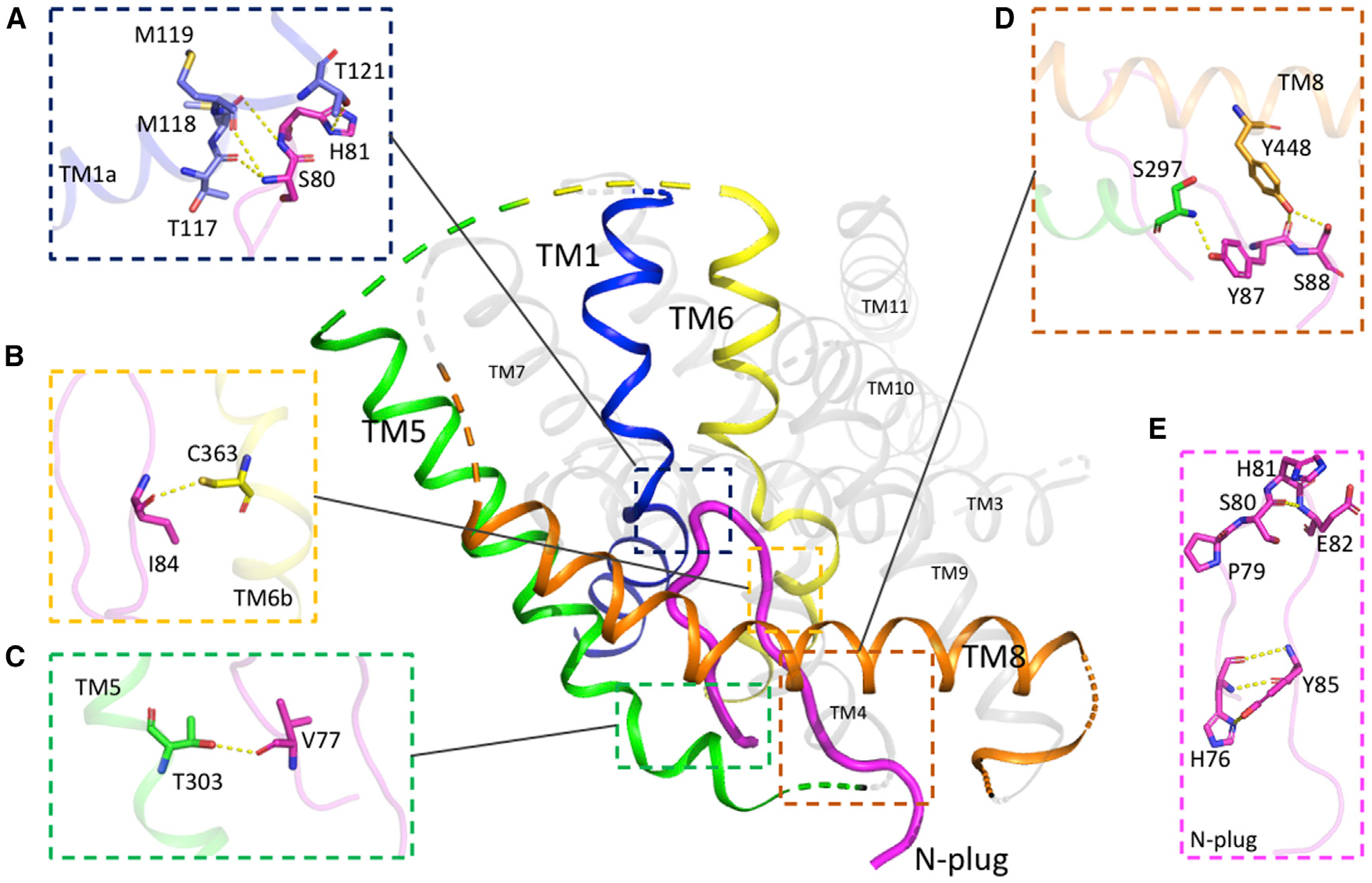
Inter- and Intra-Domain Interactions of the N-Plug Inside SLC38A9 (A–D) The N-plug interacts with the transmembrane bundle though multiple inter-domain hydrogen bonds. Residues that contribute interactions between the N-plug and TMs are highlighted in sticks and hydrogen bonds are depicted as dashed lines. (E) The folded conformation of N-plug as a β hairpin is complementarily stabilized by several intra-domain interactions.

We captured drSLC38A9 in a physiologically relevant state that we term the “N-plug inserted state.” TMs 1, 5, 6, and 8 of drSLC38A9 form a V-shaped cavity into which the N-plug inserts and is stabilized by several bonds (Figure 2). At the tapered tip on the N-plug, Ser80 and His81 are bound to the main-chain carbonyl oxygens of Thr117, Met118, and Met119 in the unwound region of TM1 (Figure 2A). Thr117, Met118, and M119 are residues known to be important for arginine uptake in humans and in drSLC38A9 (Lei et al., 2018; Wyant et al., 2017). His81 further stabilizes the tip region of the N-plug through a hydrogen bond between its imidazole side chain and Thr121 (Figure 2A). Likewise, the main-chain carbonyl oxygen of Ile 84 is bound to Cys363 on TM6 (Figure 2B). At this juncture, the N-plug is jammed in between the two essential TMs 1 and 6, where it would probably prevent the TM domain from transitioning to an alternate state for transport. At the N terminus of the N-plug, the flanking residues are anchored against TM5 through a hydrogen bond formed between the main-chain carbonyl oxygens of Val77 and the side-chain hydroxyl group of Thr303 (Figure 2C). At the C terminus of the N-plug, the Tyr-Ser pairs involving Tyr87, Tyr448 and Ser88, Ser297 also stabilize the interaction by hydrogen bonds (Figure 2D). All residues that participate in the inter-domain interactions are conserved across species as indicated in the sequence alignments (Figure S3), suggesting that this interaction is evolutionarily conserved and likely plays an important functional role. The β hairpin-like structure of the N-plug is also self-stabilized by several hydrogen bonds between Ser80 and Glu82, His76, and Tyr85, which fasten the two ends of the N-plug together (Figure 2E). Structural modeling by PEP-FOLD (Shen et al., 2014; Thevenet et al., 2012) indicated that the β hairpin motif would be converted to an α-helical fragment should these residues be changed to alanine (Figure S4).

**Figure 3.**
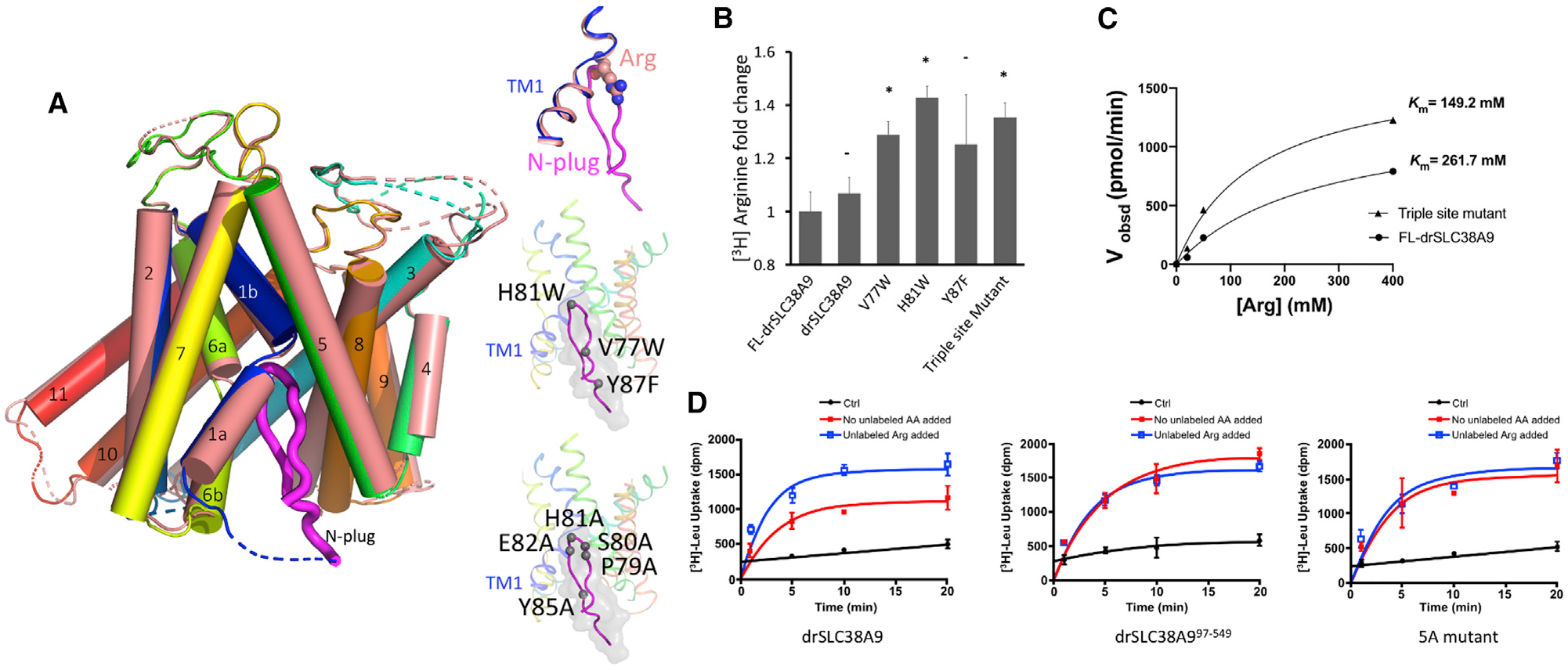
The N-Plug is Essential for Arginine-Enhanced Transport of Leucine by drSLC38A9 (A) Superposition of substrate binding site of the arginine-bound state (PDB: 6C08) with the N-plug inserted state of drSLC38A9. TM1s of two different states are shown in pink and blue. Atoms of arginine molecule are depicted as spheres while the N-plug is in magenta. Top inset: close-up view of Arg clashed on top of the N-plug in the superposition. Middle inset: positions of triple site mutations on the N-plug. Bottom inset: positions of 5A mutations on the N-plug. (B) [^3^H]Arginine steady-state uptake by drSLC38A9 variants. Fold changes are relative to the uptake by full-length drSLC38A9, and bar graphs show mean ± SEM (n = 3, *p < 0.1). (C) Michaelis-Menten plot for steady-state kinetic analysis of arginine uptake by triple site mutant and full-length drSLC38A9. The experiment was repeated more than three times with similar results and a representative one is shown. *k*_cat_ values for FL-drSLC38A9 and the triple site mutant are 79 and 60 min^−1^. (D) Adding 200 μM unlabeled arginine boosts leucine transport by drSLC38A9 in proteoliposomes (left). Either deletion (middle) or mutation of the N-plug (right) interferes with the arginine enhancement of leucine transport. The mutant proteins show similar transport capacity for leucine regardless of whether arginine was added.

Functional assays in reconstituted liposomes indicated that the N-plug plays an important role in modulating arginine uptake. An overlay of the N-plug bound structure and the arginine-bound structure of drSLC38A9 indicated that the same set of backbone atoms are used for binding the N-plug and the arginine molecule (Figure 3A). This superposition suggests that, in the presence of arginine, the N-terminal plug may not occupy the binding site, but that in the absence of arginine it would be free to insert and bind.

Several drSLC38A9 variants were generated to test the influence of the N-plug on arginine uptake, single site mutants (V77W, H81W, Y87F) and a triple site mutant (V77W + H81W + Y87F). These mutations were chosen to interfere with the binding of the N-plug in the arginine binding site of drSLC38A9. While the construct studied structurally here and the wild-type drSLC38A9 transport arginine with similar rates, all mutant SLC38A9 displayed significantly higher arginine transport efficiency (Figure 3B). These results suggest that the N-plug plays an inhibitory role to downregulate the transport of arginine by drSLC38A9. Consistent with this postulate, the triple site mutant has a 2-fold decrease in *K*_m_ for arginine without a significant change in *V*_max_ (Figure 3C). These results indicate that the insertion of the N-plug into the arginine binding pocket of drSLC38A9 is physiologically relevant and not simply a crystallization artifact. It remains to be discovered why such a mechanism is required to modulate the arginine transport by this transporter.

SLC38A9 has higher affinity toward leucine than arginine, although the transport of leucine is largely facilitated by the presence of arginine (Wyant et al., 2017). Uptake studies performed here with drSLC38A9 corroborate the previous findings using the human protein (Figure 3D). Leucine uptake was significantly higher in the presence of supplemented arginine than without. Is it possible, therefore, that in the presence of arginine the N-terminal plug could play an important role in facilitating leucine transport?

To examine whether the N-terminal plug plays an important role in facilitating leucine transport, we used two drSLC38A9 variants, one without an N-plug (drSLC38A^97−549^) and the other with five-point mutations on the N-plug (P79A, S80A, H81A, E82A, and Y85A). From the results of leucine uptake by drSLC38A9, the arginine-enhanced transport of leucine is reflected as increased uptake of [^3^H]leucine when the buffer was supplemented with arginine. This characteristic of arginine-enhanced leucine transport was lost when the N-plug was eliminated, or its structure altered by mutation. Only the drSLC38A9 with an intact N-terminal plug in its native β hairpin structure showed the characteristic of enhanced leucine uptake in the presence of supplemented arginine (Figure 3D).

It is known that the N-terminal domain of SLC38A9 can bind to, and activate, the Rag GTPase complex (Wang et al., 2015). Moreover, it was shown that the N-terminal fragment of human SLC38A9 (hSLC38A9) was sufficient and required to bind the Ragulator-Rag GTPase complex (Wang et al., 2015). The binding of Rag GTPases and the human SLC38A9 involves the 85PDH87 motif (Rebsamen et al., 2015), Pro 85 and Pro 90 (Wang et al., 2015), corresponding to a conserved region on the N-plug in drSLC38A9 (Figure S3). To probe the N-plug interaction with the Rag GTPases in drSLC38A9, we co-purified the zebrafish Rag GTPase complex (drRagA and drRagC) with two N-terminal fragments of drSLC38A9 by size-exclusion chromatography. The first fragment (residues 1–96) contained the entire N terminus (called drSLC38A9-N.1), while in the second fragment (residues 1–70) the N-plug was deleted (called drSLC38A9-N.2). Fractions from size-exclusion chromatography were collected and analyzed by SDS-PAGE (Figure S5). Contrary to fragment drSLC38A9-N.1, which maintains the N-terminal domain in its entirety, the N-plug-deleted construct, drSLC38A9-N.2, did not associate with the Rag GTPase complex (Figure S5). These results clearly demonstrated that the interaction between the zebrafish SLC38A9 N terminus and the zebrafish Rag GTPase recapitulate the experiments reported previously using human proteins (Rebsamen et al., 2015; Wang et al., 2015): the same region of the N-plug of drSLC38A9 is essential for binding with the Rag GTPase complex.

## DISCUSSION

In considering our recently determined structure of SLC38A9 with arginine bound, and the current structure without arginine but with the N-plug inserted into the arginine binding site, we have now revealed that SLC38A9 has at least two distinct conformations of the N terminus. The first is when the N-plug is bound snugly in the arginine binding site (in the absence of arginine, low luminal arginine state) and the second is where the N-terminal plug is released and the substrate-binding site is occupied by arginine (in the presence of arginine, high luminal arginine state). The vestibule into which the N-terminal plug inserts measures ~20Å in diameter. A recently determined crystal structure of Rag GTPase-regulator (De Araujo et al., 2017; Su et al., 2017; Yonehara et al., 2017) indicated that the GTPase-regulator is far too large to fit inside the vestibule of SLC38A9 suggesting that the N-plug must exit the transceptor for binding the Rag GTPase. Together, these data suggest a mechanism by which SLC38A9 can act as a receptor to signal the activation of Rag GTPase and therefore of mTORC1 in the presence of arginine.

**Figure 4.**
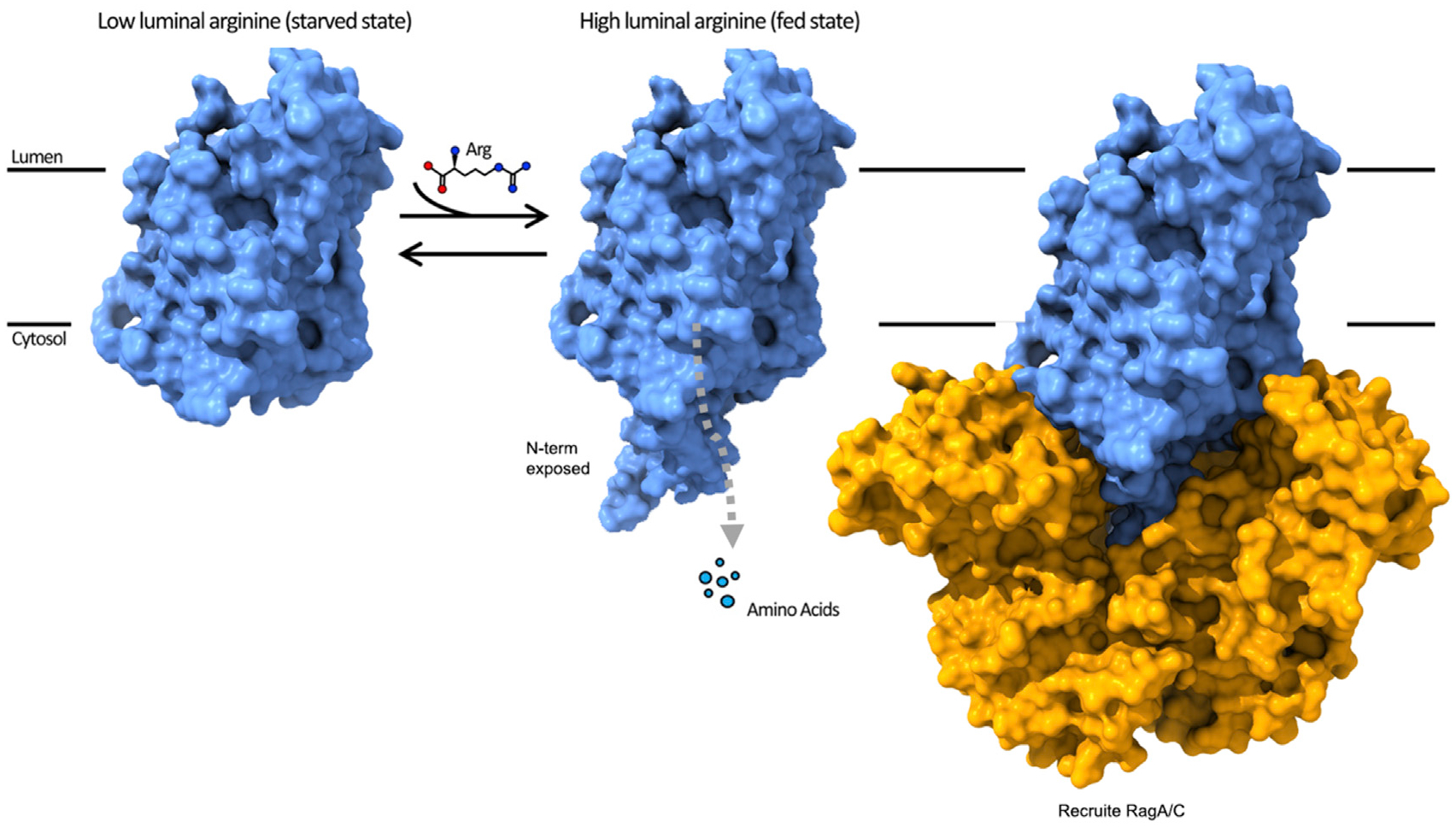
Ball-and-Chain Model of SLC38A9 for mTORC1 Activation and Amino Acid Transport At low luminal arginine, the N-plug domain dynamically samples both the inserted and released states as an equilibrium. As the concentration of luminal arginine increases, arginine molecules enter the substrate binding site of the transporter from the luminal side and the N-plug remains in the exposed state while arginine transport takes place to the cytoplasm. In the released state the N-plug could both trigger the efflux of other luminal amino acids, such as leucine, and interact with the Rag GTPases to activate the mTORC1 signaling pathways.

Thus, we propose a ball-and-chain model (Figure 4). At low luminal arginine concentrations, two conformational states could be at an equilibrium where the N-terminal plug is inserted or released from the arginine binding site of SLC38A9 at equal rates. When the equilibrium shifts to the high luminal arginine state, an arginine molecule will occupy the binding site of SLC38A9 for transport and the N-terminal plug would spend more time in the released state as long as arginine occupies the binding site. As a result, the N-terminal plug becomes available for binding to the Rag GTPase complex, which in turn could activate mTORC1. Moreover, the release of the N-terminal plug from the helical bundle of SLC38A9 will also facilitate the efflux of other essential amino acids, which simultaneously increases the cytosolic concentration of amino acids and synergistically activates mTORC1 through other cytosolic sensors.

While this study provides evidence on the function of SLC38A9 as a transporter and sensor for amino acids, it remains unclear how the N-terminal domain associates with the Rag GTPase complex at the lysosomal surface. Recently, a cryoelectron microscopic structure of the RagA/C-Ragulator in complex with the N-terminal peptide of SLC38A9 demonstrated that the human SLC38A9 is structurally incorporated in the GTPase for its nucleotide exchange activity (Fromm et al., 2020). This study agrees with our proposed ball-and-chain model of SLC38A9 for amino acid-mediated signaling and mTORC1 activation. Likewise, it is still not known what the open-to-lumen conformation of the transporter looks like and we are only now beginning to understand the dynamics of the N-plug insertion and release and its effect on arginine and leucine transport. For example, is it sufficient to simply bind arginine for signaling to occur through the N-plug or is transport of arginine required? Is it possible that additional arginine binding sites exist on SLC38A9 other than the substrate binding site describe here? Future studies must delve into these important open questions but with the above proposed ball-and-chain model for signaling, new biochemical assays can be designed and tested.

## STAR★METHODS

### RESOURCE AVAILABILITY

#### Lead Contact

Further information and requests for resources and reagents should be directed to and will be fulfilled by the Lead Contact, Tamir Gonen (tgonen@g.ucla.edu).

#### Materials Availability

There are restrictions to the availability of monoclonal antibody 11D3 due to the lack of an external centralized repository for its distribution and our need to maintain the stock. We are glad to share monoclonal antibody 11D3 with reasonable compensation by requestor for its processing and shipping.

#### Data and Code Availability

The atomic coordinates of drSLC38A9 with N-plug has been deposited into the Protein Data Bank (PDB: 7KGV).

### EXPERIMENTAL MODEL AND SUBJECT DETAILS

#### Cell Line

Spodoptera frugiperda Sf-9.

#### Culture Conditions for In Vitro Systems

Cells were grown in ESF 921 Insect Cell Culture Medium in flasks at 27°C.

### METHOD DETAILS

#### Protein Expression and Purification

The protein used in this study is drSLC38A9^71−549^ referred to as drSLC38A9 in this manuscript. The gene of wild-type SLC38A9 (NP_001073468.1) from *Danio rerio* and its mutants were synthesized and then sub-cloned into a pFastbac1 vector containing an octa-histidine tag with a thrombin-cleavage site at the N-terminus. drSLC38A9 protein and its variants were overexpressed in *Spodoptera frugiperda* Sf-9 insect cells following the protocol of Bac-to-Bac Baculovirus Expression System (Invitrogen). Cells were harvested at 60 hours after infection and homogenized in the low salt buffer containing 20 mM Tris pH 8.0, 150 mM NaCl supplemented with cOmplete Protease Inhibitor Cocktail (Roche). The lysate was collected and ultra-centrifuged at 130,000×*g* for 1 hour. Pelleted membrane was then resuspended and washed with the high salt buffer containing 1.0 M NaCl and 20 mM Tris (8.0) followed by ultracentrifugation. The pellets were resuspended in the low salt buffer, frozen in liquid nitrogen and stored in −80°C until further use.

To purify drSLC38A9 protein and its variants, membrane fraction was thawed and solubilized with 2% n-dodecyl-b-D-maltopyranoside (DDM, Anatrace) in 20 mM Tris pH 8.0, 500 mM NaCl, 5% glycerol, and 0.2% Cholesteryl Hemisuccinate Tris Salt (CHS, Anatrace) for 4 hours at 4°C. Following another ultra-centrifugation at 130,000×*g* for 1 hour, the supernatant was loaded onto TALON Metal Affinity Resin (Clontech) and incubated at 4°C overnight. The resins were washed by 5× column volumes of 50 mM imidazole, 20 mM Tris pH 8, 500 mM NaCl, 0.1% DDM before equilibration in 20 mM Tris pH 8.0, 500 mM NaCl, 0.4% decyl-b-D-maltoside (DM) and 0.02% DDM. The N-terminal octa-histidine tag was removed by in-column thrombin digestion overnight at enzyme:protein molar ratio of 1:1000. The cleaved drSLC38A9 proteins collected in flow-through were then flash-frozen in liquid nitrogen and stored in −80°C until use.

#### Fab Fragments Production

Fab fragments were produced at Monoclonal Antibody Core of Vaccine and Gene Therapy Institute, OHSU. Mouse IgG monoclonal antibodies against drSLC38A9 were raised by standard protocol (Harlow and Lane, 1988) using purified protein in the buffer containing 20 mM Tris pH 8.0, 150 mM NaCl, 0.02% DDM, 0.002% CHS as antigen. Western blot and native-to-denature ELISA assays (Lim et al., 2011) were performed to assess the binding affinity and specificity of the antibodies generated from hybridoma cell lines. Several monoclonal antibodies showing high binding affinity and specificity to conformational epitope were then selected and purified from the hybridoma supernatants. Fab fragments were generated by Papain (Thermo Fisher Scientific) digestion and purified by Protein A affinity chromatography (GE Healthcare) in 20 mM Sodium phosphates pH 8.0, 150 mM NaCl.

#### Purification of drSLC38A9-Fab Complexes for Crystallization

Purified drSLC38A9 proteins was mixed with excess Fab fragments at a molar ratio of 1:2 for 2 hours, and the mixture was subjected to gel filtration (Superdex 200 Increase 10/300 GL, GE Healthcare) in the buffer containing 20 mM Tris-HCl pH 8.0, 150 mM NaCl and 0.2% DM. The peak fractions containing appropriate drSLC38A9-Fab complexes were then pooled and concentrated to 5 mg/mL for crystallization.

#### Crystallization

Crystallization was carried out by hanging-drop vapor diffusion at 4°C. Initial hits of drSLC38A9 were identified in multiple conditions containing PEG 400. However, these crystals gave anisotropic diffraction to ~6 Å. Well-diffracting crystals were only obtained when drSLC38A9 was co-crystallized as a complex with Fab fragment prepared from hybridoma cell line 11D3 (IgG2a, kappa) at 5 mg/mL mixed 1:1 with drop solution containing 30% PEG 400, 100 mM ADA pH 6.0 and 350 mM Li_2_SO_4_.

#### Data Collection and Structure Determination

Before data collection, crystals were soaked in a cryoprotectant buffer containing 30% PEG 400 in the same crystallizing solution for 1 min, and rapidly frozen in liquid nitrogen. All diffraction data for drSLC38A9-Fab complex were collected at 100K using synchrotron radiation at the Advanced Photon Source (NE-CAT 24-ID-C and 24-ID-E). Diffraction data indexing, integration and scaling were performed with the online RAPD system and the CCP4 suite (Winn et al., 2011). Data collection statistics, phasing and refinement are given in Table 1. Molecular replacement using Phaser (McCoy et al., 2007) was able to place two copies of Fab fragment (PDB: 1F8T) in native datasets. Helices of drSLC38A9 were manually placed in the density-modified map and extended within Coot (Emsley et al., 2010) according to the reference model of ΔN-drSLC38A9-Fab complex (PDB: 6C08). Subsequent cycles of density modifications, model building and refinement were carried out in Phenix (Adams et al., 2010; Zwart et al., 2008) and Coot until structure completion (Figure S2). The Ramachandran analyses of final structures were performed using Molprobity (Chen et al., 2010). The model has been deposited into the PDB (PDB: 7KGV).

#### Proteoliposome Reconstitution and Arginine Uptake Assay

The full-length drSLC38A9, three single-mutants (V77W; H81W; Y87F), and triple-mutant (V77W, H81W, and Y87F) proteins were expressed and purified as described above. Chloroform-dissolved chicken egg phosphatidylcholine (egg-PC, Avanti Polar Lipids) was evaporated using dry nitrogen to yield a lipid film in a small glass vial and further dried under vacuum overnight. The lipids were hydrated in inside buffer (20 mM Mes pH 5.0, 90 mM KCl, 10 mM NaCl) at 25 mg/mL by vortex for 3 minutes and then aged in room temperature for 1 hour. Liposomes were clarified by 5 rounds of freezing and thawing in liquid nitrogen and extruded through a 100 nm membrane with 21 passes (Milipore). The liposomes were pre-incubated with 1% n-octyl-β-D-glucoside (β-OG) and 1 mM DDT for 1 hour at 4°C before protein reconstitution. Purified full-length drSLC38A9 and variants were incorporated at a 1:80 (w/w) ratio into destabilized liposomes for 1 hour in the 4°C rotator. Glycerol-supplemented protein buffer was used in lieu of drSLC38A9 protein in liposome-only control groups. The detergents were removed by incubation overnight with 200 mg per reaction Bio-Beads, and the proteoliposomes were further incubated with 40 mg per reaction fresh Bio-Beads for an additional hour. The proteoliposomes and liposome-only controls were collected using ultracentrifuge at 100,000×g for 30 minutes at 4°C and then resuspended in outside buffer (20 mM Tris pH 7.4, 100 mM NaCl) to final lipid concentration of 32 μg/μL.

Transport reactions were initiated by adding 0.5 μM L-[3H]-arginine (American Radiolabeled Chemicals, Inc) to 50 μL of proteoliposomes. Assays of Liposome-only controls were carried out in parallel to experimental groups as negative controls. All buffers were chilled and assays were performed at room temperature. For time-course uptake assay, at various time points, proteoliposomes were filtered, washed by 5mL of ice-cold wash buffer (outside buffer with 10 mM unlabeled L-arginine), and collected on 0.22 μm nitrocellulose membranes (Millipore) which had been pre-wet by washing buffer. After washing, each filter was dried by vacuum for exactly 1 minute and transferred into a glass vial with 10 mL scintillation fluid for counting. Measurements at 5 minutes of the arginine uptake were used to establish the transport comparisons between various constructs of drSLC38A9, normalized to that of the full-length wildtype drSLC38A9. Non-specific adsorptions of L-[3H]-arginine by liposomes-only controls were subtracted from experimental measurements.

The measurements of Km and kcat were performed in the presence of unlabeled L-arginine at the indicated concentrations supplemented with outside buffer, together with the same concentration of L-[3H]-arginine at 0.5 μM. All outside buffers (with different concentrations of unlabeled arginine) were adjusted to pH 7.4. The uptake of L-[3H]-arginine was stopped at 5 minutes when the transport activity still remained linear. The experiment was repeated more than three times with similar results and a representative one is shown.

#### Proteoliposome Reconstitution and Leucine Uptake Assay

The full-length drSLC38A9 and two variants, N-terminal deletion (truncate N-terminus from Met 1 to Val 96) and 5A (P79A, S80A, H81A, E82A, and Y85A) mutant protein, were expressed and purified as described above. Liposomes were prepared using a 3:1 ratio of *E. coli* total lipid extract (Avanti Polar Lipids) to chicken egg phosphatidylcholine (egg-PC, Avanti Polar Lipids) at 20 mg/mL in assay buffer (20mM MES pH 5.0, 150mM NaCl and 1mM DTT). An extruder with pore size of 0.4 μm was used to obtain unilamellar vesicles. Triton X-100 was then added to the extruded liposomes at 10:1 (w:w) lipid:detergent ratio. Purified wild-type drSLC38A9 and variants were reconstituted at a 1:200 (w/w) ratio in destabilized liposomes and excess detergent was removed by SM2 Bio-Beads (Bio-Rad) at 4°C overnight. Next day, proteoliposomes were collected, aliquoted and frozen at −80°C for storage until needed.

Transport reactions were initiated by adding [^3^H]-labeled amino acids (American Radiolabeled Chemicals) to 50 μL of 10-fold diluted proteoliposomes (total of 0.5 μg protein) to final concentration of 0.5 μM at room temperature. As controls, non-specific uptake was assessed by using protein-free liposomes under identical conditions in parallel to experimental groups. At various time points, reactions were stopped by quenching the samples with 5 mL assay buffer followed by rapid filtration through 0.22μm membrane filter (GSWP02500, MilliporeSigma) to remove excess radioligands. The filter was then washed three times with 5 mL assay buffer, suspended in 10 mL of scintillation fluid and quantified by scintillation counting. A time course profile indicates that the retained radio-ligands reached saturation after 10 min. Measurements at various time points of the uptake were plotted to establish the transport comparisons between various constructs of drSLC38A9. All experiment and control groups were repeated two to three times.

#### Co-purification of Zebrafish Rag GTPase Complex with N-Terminal Fragment of drSLC38A9

The synthesized cDNA encoding RagA (UniProtKB - Q7ZUI2) and RagC (UniProtKB - F1Q665) from *Danio rerio* were cloned into pFastBac Dual vector. The Rag GTPase complex were overexpressed in *Spodoptera frugiperda* Sf-9 insect cells, which was harvested at 48 hours post-infection. Cell pellets were resuspended in lysis buffer containing 20 mM Tris pH 8.0, 150 mM NaCl. 30 homogenizing cycles were then carried out to break cells on ice, followed by a centrifugation at 130,000×*g* for 30 mins. The supernatant was incubated with Ni-NTA Agarose (QIGEN) for 2 hours at 4°C. The resins were then washed with 5× column volumes of wash buffer containing 50mM Imidazole, 20 mM Tris pH 8.0, 150 mM NaCl. The protein was eluted by elution buffer containing 300 mM imidazole, 20 mM Tris pH 8.0, 150 mM NaCl, and then applied to gel filtration (Superdex 200 Increase 10/300 GL, GE Healthcare) in 20 mM Tris pH 8.0, 150 mM NaCl. The peak fractions were collected for further analysis.

To enhance solubility and stability, the N-terminal fragments of drSLC38A9 were fused with GB1 domain-tag (Cheng and Patel, 2004). drSLC38A9-N.1 is from Met 1 to Val 96, and drSLC38A9-N.2 is from Met 1 to Leu 70. The fusion proteins were overexpressed in *E. coli* BL21 (DE3) at 16°C for overnight with 0.2 mM isopropyl-β-D-thiogalactopyranoside (IPTG) as inducer. Then, the cells were harvested, homogenized in a lysis buffer containing 20mM Tris pH 8.0 and 150mM NaCl, and disrupted using a Microfluidizer (Microfluidics Corporation) with 3 passes at 15,000 p.s.i., followed by a centrifugation for 30 mins to remove cell debris. The supernatant was then loaded onto Ni-NTA Agarose and purified as above.

The purified Rag GTPase complex was mixed with excess GB1-drSLC38A9-N.x fragment at a molar ration of 1:2 for 1 hour, and the mixture was then subjected to gel filtration (Superdex 200 Increase 10/300 GL, GE Healthcare) in the buffer containing 20 mM Tris pH 8.0, 150 mM NaCl. SDS-PAGE and Coomassie blue staining was used to analyze the size exclusion chromatography elution profile.

All figures in this paper were prepared with PyMOL v1.8.6.0 (Schrodinger LLC, 2015). Figure S3 was prepared using the program Clustal Omega (Sievers et al., 2011) for alignments and ESPript 3.0 (Robert and Gouet, 2014) for styling.

### QUANTIFICATION AND STATISTICAL ANALYSIS

All statistical analyses were performed in Microsoft Excel and GraphPad Prism.

The statistical details of arginine and leucine uptake assay can be found in main text and figure legends. The significance was determined by unpaired t-test in Figure 3B.

## Supplementary Material

1

## Figures and Tables

**Table 1.** Data Collection and Refinement Statistics

	S38A9-Fab^a^
Data Collection	
Space group	P 1 21 1
Cell dimensions	
a, b, c (Å)	157.79, 82.51, 158.59
α, β, γ (°)	90.00, 106.02, 90.00
R_pim_^c^	0.084 (1.071)
Mean I/σ(I)	6.5 (1.0)
Completeness (%)	99.9 (99.8)
Redundancy	11.8 (6.1)
CC½	0.989 (0.350)
Refinement	
Resolution (Å)	49.37–3.40 (3.50–3.40)^b^
No. of reflections	54,396 (4,463)
R_work_/R_free_	0.251/0.284
No. of non-hydrogen atoms	
Protein	12,613
Average B factors	
Protein	110.3
RMSD	
Bond lengths (Å)	0.011
Bond angles (°)	1.325
Ramachandran plot (%)	
Favored	90.27
Outliers	1.58

RMSD, root-mean-square deviation.

a Four crystals were used to collect diffraction datasets which were processed, scaled, and merged using RAPD and AIMLESS.

b Values in parentheses are for the highest-resolution shell used in model refinement (3.50–3.40Å_)._

c R_pim_ is a measure of the quality of the data after averaging the multiple measurements and R_pim_ = Σ_hkl_ [n/(n − 1)]^1/2^ Σ_i_|I_i_(hkl) − <I(hkl)> |/Σ_hkl_ Σ_i_ I_i_(hkl), where n is the multiplicity, I_i_ is the intensity of the ith observation, <I> is the mean intensity of the reflection and the summations extend over all unique reflections (hkl) and all equivalents (i), respectively (Weiss, 2001).

**Table:** KEY RESOURCES TABLE

REAGENT or RESOURCE	SOURCE	IDENTIFIER
Antibodies		
Monoclonal antibody clone 11D3	This paper	N/A
Deposited Data		
Arginine-bound drSLC38A9	Lei et al. (2018)	PDB: 6C08
N-plug inserted drSLC38A9	This paper	PDB: 7KGV
Experimental Models: Organisms/Strains		
*Spodoptera frugiperda* Sf-9 insect cells	Expression Systems, LLC	Cat#94–001F
Oligonucleotides		
FL-drSLC38A9	CCATCACCACCACCATCACCATCACCTCGTGCCCAGGGGATCCATGGACG AGGATTCTAAGCCGCTGCTCGGATCAGTGCCTACCGGTGACTACTACACT GACTCCCTGGACCCTAAACAAAGGAGGCCATTCCACGTGGAGCCAAGGA ACATCGTCGGCGAGGATGTTCAAGAACGTGTGAGCGCTGAAGCTGCTGT GCTGTCCAGCAGGGTCCACTACTACTCTCGTCTCACCGGATCTTCAGAC AGGTTGCTGGCACCTCCCGATCACGTGATTCCGTCTCATGAAGACATCTA CATTTACTCACCACTGGGCACCGCATTCAAGGTCCAGGGTGGCGATAGTC CAATCAAAAACCCGTCGATCGTTACTATTTTCGCGATCTGGAACACCATGA TGGGAACTAGTATTCTGTCGATCCCTTGGGGTATCAAGCAAGCTGGATTCA CCCTCGGTATCATTATCATTGTGCTGATGGGACTCTTGACTCTCTACTGCT GTTACCGCGTTCTGAAGTCCACAAAAAGCATCCCTTACGTGGACACGAGC GATTGGGAGTTCCCCGACGTCTGCAAGTACTACTTCGGAGGTTTCGGCAAA TGGAGTTCGCTGGTCTTCTCTCTCGTTTCATTGATCGGAGCTATGGTGGTCT ACTGGGTCCTCATGTCAAACTTCTTGTTCAACACTGGCAAGTTCATTTTCAA CTACGTTCACAACGTGCAGACGAGTGACGCTTTCGGTACACAGGGCACGGAG CGTGTTATCTGTCCTTACCCCGACGTGGACCCTCACGGCCAGTCCAGCAC CAGTTTGTACTCCGGCAGCGACCAGTCGACTGGACTGGAATTCGATCATTG GTGGTCTAAGACAAACACGATCCCCTTCTACCTGATTCTGCTCTTGCTGCCA CTCTTGAACTTCAGGTCTGCTTCATTCTTCGCCAGATTCACATTCCTGGGAA CGATTTCAGTCATCTACCTGATTTTCCTCGTTACTTACAAAGCCATCCAGCT GGGTTTCCACCTCGAGTTCCATTGGTTCGACTCTTCAATGTTCTTCGTGCCC GAATTCAGGACATTGTTCCCACAGCTGTCCGGCGTCCTCACGTTGGCTTTCT TCATCCACAACTGCATCATTACACTGATGAAGAACAACAAACATCAAGA GAACAACGTTAGAGACCTGAGCCTCGCCTACCTGCTCGTGGGTTTGACCT ACCTGTACGTCGGCGTTCTCATCTTCGCAGCGTTCCCAAGTCCACCGTTG TCGAAGGAGTGTATCGAACCGAACTTCCTCGACAACTTCCCTAGTTCGGAT ATTTTGGTGTTCGTCGCTCGCACATTCTTGCTGTTCCAGATGACCACTGTG TACCCACTCTTGGGTTACCTGGTTCGTGTGCAGCTCATGGGCCAAATCTTC GGAAACCACTACCCTGGCTTCTTGCATGTCTTCGTTCTGAACGTGTTCGT TGTGGGCGCAGGAGTCCTGATGGCGAGGTTCTACCCCAACATCGGCTCCA TCATTAGATACAGCGGTGCTCTCTGCGGCTTGGCCCTGGTGTTCGTCCTC CCATCCTTGATTCACATGGTGAGCTTGAAGAGGAGAGGAGAACTGCGTTG GACCTCCACTCTCTTCCATGGTTTCTTGATCCTGCTCGGCGTCGCTAACT TGCTGGGACAATTCTTCATGTAATAAGCTT	GenScript
drSLC38A9, V77W	CCATCACCACCACCATCACCATCACCTCGTGCCCAGGGGATCCATGGACGAGGATTCTAAG CCGCTGCTCGGATCAGTGCCTACCGGTGACTACTACACTGACTCCCTGGACCCTAAACAAA GGAGGCCATTCCACGTGGAGCCAAGGAACATCGTCGGCGAGGATGTTCAAGAACGTGTGAG CGCTGAAGCTGCTGTGCTGTCCAGCAGGGTCCACTACTACTCTCGTCTCACCGGATCTTCA GACAGGTTGCTGGCACCTCCCGATCACTGGATTCCGTCTCATGAAGACATCTACATTTACT CACCACTGGGCACCGCATTCAAGGTCCAGGGTGGCGATAGTCCAATCAAAAACCCGTCGA TCGTTACTATTTTCGCGATCTGGAACACCATGATGGGAACTAGTATTCTGTCGATCCCTTG GGGTATCAAGCAAGCTGGATTCACCCTCGGTATCATTATCATTGTGCTGATGGGACTCTT GACTCTCTACTGCTGTTACCGCGTTCTGAAGTCCACAAAAAGCATCCCTTACGTGGACAC GAGCGATTGGGAGTTCCCCGACGTCTGCAAGTACTACTTCGGAGGTTTCGGCAAATGGA GTTCGCTGGTCTTCTCTCTCGTTTCATTGATCGGAGCTATGGTGGTCTACTGGGTCCTCAT GTCAAACTTCTTGTTCAACACTGGCAAGTTCATTTTCAACTACGTTCACAACGTGCAGACGA GTGACGCTTTCGGTACACAGGGCACGGAGCGTGTTATCTGTCCTTACCCCGACGTGGA CCCTCACGGCCAGTCCAGCACCAGTTTGTACTCCGGCAGCGACCAGTCGACTGGACTGG AATTCGATCATTGGTGGTCTAAGACAAACACGATCCCCTTCTACCTGATTCTGCTCTTGCT GCCACTCTTGAACTTCAGGTCTGCTTCATTCTTCGCCAGATTCACATTCCTGGGAACGAT TTCAGTCATCTACCTGATTTTCCTCGTTACTTACAAAGCCATCCAGCTGGGTTTCCACCTC GAGTTCCATTGGTTCGACTCTTCAATGTTCTTCGTGCCCGAATTCAGGACATTGTTCCC ACAGCTGTCCGGCGTCCTCACGTTGGCTTTCTTCATCCACAACTGCATCATTACACTGAT GAAGAACAACAAACATCAAGAGAACAACGTTAGAGACCTGAGCCTCGCCTACCTGCTC GTGGGTTTGACCTACCTGTACGTCGGCGTTCTCATCTTCGCAGCGTTCCCAAGTCCACC GTTGTCGAAGGAGTGTATCGAACCGAACTTCCTCGACAACTTCCCTAGTTCGGATATT TTGGTGTTCGTCGCTCGCACATTCTTGCTGTTCCAGATGACCACTGTGTACCCA CTCTTGGGTTACCTGGTTCGTGTGCAGCTCATGGGCCAAATCTTCGGAAACCAC TACCCTGGCTTCTTGCATGTCTTCGTTCTGAACGTGTTCGTTGTGGGCGCAGGA GTCCTGATGGCGAGGTTCTACCCCAACATCGGCTCCATCATTAGATACAGCGG TGCTCTCTGCGGCTTGGCCCTGGTGTTCGTCCTCCCATCCTTGATTCACATGGT GAGCTTGAAGAGGAGAGGAGAACTGCGTTGGACCTCCACTCTCTTCCATGGTTT CTTGATCCTGCTCGGCGTCGCTAACTTGCTGGGACAATTCTTCATGTAATAAGCTT	GenScript
drSLC38A9, triple-mutant (V77W, H81W, and Y87F)	CCATCACCACCACCATCACCATCACCTCGTGCCCAGGGGATCCATGGACGAGG ATTCTAAGCCGCTGCTCGGATCAGTGCCTACCGGTGACTACTACACTGACTCCC TGGACCCTAAACAAAGGAGGCCATTCCACGTGGAGCCAAGGAACATCGTCGG CGAGGATGTTCAAGAACGTGTGAGCGCTGAAGCTGCTGTGCTGTCCAGCAGGG TCCACTACTACTCTCGTCTCACCGGATCTTCAGACAGGTTGCTGGCACCTCCCGA TCACTGGATTCCGTCTTGGGAAGACATCTACATTTTCTCACCACTGGGCACCGCA TTCAAGGTCCAGGGTGGCGATAGTCCAATCAAAAACCCGTCGATCGTTACTATTT TCGCGATCTGGAACACCATGATGGGAACTAGTATTCTGTCGATCCCTTGGGGTA TCAAGCAAGCTGGATTCACCCTCGGTATCATTATCATTGTGCTGATGGGACTCTT GACTCTCTACTGCTGTTACCGCGTTCTGAAGTCCACAAAAAGCATCCCTTACG TGGACACGAGCGATTGGGAGTTCCCCGACGTCTGCAAGTACTACTTCGGAGG TTTCGGCAAATGGAGTTCGCTGGTCTTCTCTCTCGTTTCATTGATCGGAGCTATGG TGGTCTACTGGGTCCTCATGTCAAACTTCTTGTTCAACACTGGCAAGTTCATTTTCAA CTACGTTCACAACGTGCAGACGAGTGACGCTTTCGGTACACAGGGCACGGAGCGT GTTATCTGTCCTTACCCCGACGTGGACCCTCACGGCCAGTCCAGCACCAGTTTGTA CTCCGGCAGCGACCAGTCGACTGGACTGGAATTCGATCATTGGTGGTCTAAGAC AAACACGATCCCCTTCTACCTGATTCTGCTCTTGCTGCCACTCTTGAACTTCAGGTC TGCTTCATTCTTCGCCAGATTCACATTCCTGGGAACGATTTCAGTCATCTACCTGA TTTTCCTCGTTACTTACAAAGCCATCCAGCTGGGTTTCCACCTCGAGTTCCATTGG TTCGACTCTTCAATGTTCTTCGTGCCCGAATTCAGGACATTGTTCCCACAGCTGTCCG GCGTCCTCACGTTGGCTTTCTTCATCCACAACTGCATCATTACACTGATGAAGAAC AACAAACATCAAGAGAACAACGTTAGAGACCTGAGCCTCGCCTACCTGCTCGTGGG TTTGACCTACCTGTACGTCGGCGTTCTCATCTTCGCAGCGTTCCCAAGTCCACCGTTG TCGAAGGAGTGTATCGAACCGAACTTCCTCGACAACTTCCCTAGTTCGGATATTTTGG TGTTCGTCGCTCGCACATTCTTGCTGTTCCAGATGACCACTGTGTACCCACTCTTG GGTTACCTGGTTCGTGTGCAGCTCATGGGCCAAATCTTCGGAAACCACTACCCTGGCTT CTTGCATGTCTTCGTTCTGAACGTGTTCGTTGTGGGCGCAGGAGTCCTGATGGCGA GGTTCTACCCCAACATCGGCTCCATCATTAGATACAGCGGTGCTCTCTGCGGC TTGGCCCTGGTGTTCGTCCTCCCATCCTTGATTCACATGGTGAGCTTGAAGAG GAGAGGAGAACTGCGTTGGACCTCCACTCTCTTCCATGGTTTCTTGATCCTGC TCGGCGTCGCTAACTTGCTGGGACAATTCTTCATGTAATAAGCTT	GenScript
drSLC38A9, truncated N-terminus from Met 1 to Val 96	CCATCACCACCACCATCACCATCACCTCGTGCCCAGGGGATCCCAGGGTGGCGATA GTCCAATCAAAAACCCGTCGATCGTTACTATTTTCGCGATCTGGAACACCATGATGGGA ACTAGTATTCTGTCGATCCCTTGGGGTATCAAGCAAGCTGGATTCACCCTCGGTATCATT ATCATTGTGCTGATGGGACTCTTGACTCTCTACTGCTGTTACCGCGTTCTGAAGTCCACA AAAAGCATCCCTTACGTGGACACGAGCGATTGGGAGTTCCCCGACGTCTGCAAGTACTA CTTCGGAGGTTTCGGCAAATGGAGTTCGCTGGTCTTCTCTCTCGTTTCATTGATCGGAGC TATGGTGGTCTACTGGGTCCTCATGTCAAACTTCTTGTTCAACACTGGCAAGTTCATTTTC AACTACGTTCACAACGTGCAGACGAGTGACGCTTTCGGTACACAGGGCACGGAGCGTGT TATCTGTCCTTACCCCGACGTGGACCCTCACGGCCAGTCCAGCACCAGTTTGTACTCCG GCAGCGACCAGTCGACTGGACTGGAATTCGATCATTGGTGGTCTAAGACAAACACGATC CCCTTCTACCTGATTCTGCTCTTGCTGCCACTCTTGAACTTCAGGTCTGCTTCATTCTTCG CCAGATTCACATTCCTGGGAACGATTTCAGTCATCTACCTGATTTTCCTCGTTACTTACAAA GCCATCCAGCTGGGTTTCCACCTCGAGTTCCATTGGTTCGACTCTTCAATGTTCTTCGTG CCCGAATTCAGGACATTGTTCCCACAGCTGTCCGGCGTCCTCACGTTGGCTTTCTTCATC CACAACTGCATCATTACACTGATGAAGAACAACAAACATCAAGAGAACAACGTTAGAGAC CTGAGCCTCGCCTACCTGCTCGTGGGTTTGACCTACCTGTACGTCGGCGTTCTCATCTT CGCAGCGTTCCCAAGTCCACCGTTGTCGAAGGAGTGTATCGAACCGAACTTCCTCGAC AACTTCCCTAGTTCGGATATTTTGGTGTTCGTCGCTCGCACATTCTTGCTGTTCCAGATG ACCACTGTGTACCCACTCTTGGGTTACCTGGTTCGTGTGCAGCTCATGGGCCAAATCTT CGGAAACCACTACCCTGGCTTCTTGCATGTCTTCGTTCTGAACGTGTTCGTTGTGGGC GCAGGAGTCCTGATGGCGAGGTTCTACCCCAACATCGGCTCCATCATTAGATACAGCG GTGCTCTCTGCGGCTTGGCCCTGGTGTTCGTCCTCCCATCCTTGATTCACATGGTGAGC TTGAAGAGGAGAGGAGAACTGCGTTGGACCTCCACTCTCTTCCATGGTTTCTTGATCCT GCTCGGCGTCGCTAACTTGCTGGGACAATTCTTCATGTAATAAGCTT	GenScript
drSLC38A9, 5A mutant (P79A, S80A, H81A, E82A, and Y85A)	CCATCACCACCACCATCACCATCACCTCGTGCCCAGGGGATCCCTGGCACCTCCCGATC ACGTGATTGCCGCTGCCGCTGACATCGCCATTTACTCACCACTGGGCACCGCATTCAAGG TCCAGGGTGGCGATAGTCCAATCAAAAACCCGTCGATCGTTACTATTTTCGCGATCTGGAA CACCATGATGGGAACTAGTATTCTGTCGATCCCTTGGGGTATCAAGCAAGCTGGATTCAC CCTCGGTATCATTATCATTGTGCTGATGGGACTCTTGACTCTCTACTGCTGTTACCGCGTT CTGAAGTCCACAAAAAGCATCCCTTACGTGGACACGAGCGATTGGGAGTTCCCCGACGTC TGCAAGTACTACTTCGGAGGTTTCGGCAAATGGAGTTCGCTGGTCTTCTCTCTCGTTTCAT TGATCGGAGCTATGGTGGTCTACTGGGTCCTCATGTCAAACTTCTTGTTCAACACTGGCA AGTTCATTTTCAACTACGTTCACAACGTGCAGACGAGTGACGCTTTCGGTACACAGGGCACG GAGCGTGTTATCTGTCCTTACCCCGACGTGGACCCTCACGGCCAGTCCAGCACCAGTTTGT ACTCCGGCAGCGACCAGTCGACTGGACTGGAATTCGATCATTGGTGGTCTAAGACAAACAC GATCCCCTTCTACCTGATTCTGCTCTTGCTGCCACTCTTGAACTTCAGGTCTGCTTCATTCTT CGCCAGATTCACATTCCTGGGAACGATTTCAGTCATCTACCTGATTTTCCTCGTTACTTACA AAGCCATCCAGCTGGGTTTCCACCTCGAGTTCCATTGGTTCGACTCTTCAATGTTCTTCG TGCCCGAATTCAGGACATTGTTCCCACAGCTGTCCGGCGTCCTCACGTTGGCTTTCTTCA TCCACAACTGCATCATTACACTGATGAAGAACAACAAACATCAAGAGAACAACGTTAGAGAC CTGAGCCTCGCCTACCTGCTCGTGGGTTTGACCTACCTGTACGTCGGCGTTCTCATCTTCG CAGCGTTCCCAAGTCCACCGTTGTCGAAGGAGTGTATCGAACCGAACTTCCTCGACAACTTC CCTAGTTCGGATATTTTGGTGTTCGTCGCTCGCACATTCTTGCTGTTCCAGATGACCACTGT GTACCCACTCTTGGGTTACCTGGTTCGTGTGCAGCTCATGGGCCAAATCTTCGGAAACCAC TACCCTGGCTTCTTGCATGTCTTCGTTCTGAACGTGTTCGTTGTGGGCGCAGGAGTCCTGA TGGCGAGGTTCTACCCCAACATCGGCTCCATCATTAGATACAGCGGTGCTCTCTGCGGC TTGGCCCTGGTGTTCGTCCTCCCATCCTTGATTCACATGGTGAGCTTGAAGAGGAGAGGAG AACTGCGTTGGACCTCCACTCTCTTCCATGGTTTCTTGATCCTGCTCGGCGTCGCTAACTT GCTGGGACAATTCTTCATGTAATAAGCTT	GenScript
drSLC38A9, truncated N-terminus from Met 1 to Leu 70	CCATCACCACCACCATCACCATCACCTCGTGCCCAGGGGATCCCTGGCACCTCCCGATCA CGTGATTCCGTCTCATGAAGACATCTACATTTACTCACCACTGGGCACCGCATTCAAGGTC CAGGGTGGCGATAGTCCAATCAAAAACCCGTCGATCGTTACTATTTTCGCGATCTGGAACA CCATGATGGGAACTAGTATTCTGTCGATCCCTTGGGGTATCAAGCAAGCTGGATTCACCCT CGGTATCATTATCATTGTGCTGATGGGACTCTTGACTCTCTACTGCTGTTACCGCGTTCTGA AGTCCACAAAAAGCATCCCTTACGTGGACACGAGCGATTGGGAGTTCCCCGACGTCTGCA AGTACTACTTCGGAGGTTTCGGCAAATGGAGTTCGCTGGTCTTCTCTCTCGTTTCATTGAT CGGAGCTATGGTGGTCTACTGGGTCCTCATGTCAAACTTCTTGTTCAACACTGGCAAGTTC ATTTTCAACTACGTTCACAACGTGCAGACGAGTGACGCTTTCGGTACACAGGGCACGGAG CGTGTTATCTGTCCTTACCCCGACGTGGACCCTCACGGCCAGTCCAGCACCAGTTTGTAC TCCGGCAGCGACCAGTCGACTGGACTGGAATTCGATCATTGGTGGTCTAAGACAAACACG ATCCCCTTCTACCTGATTCTGCTCTTGCTGCCACTCTTGAACTTCAGGTCTGCTTCATTCTTC GCCAGATTCACATTCCTGGGAACGATTTCAGTCATCTACCTGATTTTCCTCGTTACTTACAA AGCCATCCAGCTGGGTTTCCACCTCGAGTTCCATTGGTTCGACTCTTCAATGTTCTTCGTG CCCGAATTCAGGACATTGTTCCCACAGCTGTCCGGCGTCCTCACGTTGGCTTTCTTCATCC ACAACTGCATCATTACACTGATGAAGAACAACAAACATCAAGAGAACAACGTTAGAGACCT GAGCCTCGCCTACCTGCTCGTGGGTTTGACCTACCTGTACGTCGGCGTTCTCATCTTCG CAGCGTTCCCAAGTCCACCGTTGTCGAAGGAGTGTATCGAACCGAACTTCCTCGACAACT TCCCTAGTTCGGATATTTTGGTGTTCGTCGCTCGCACATTCTTGCTGTTCCAGATGACCAC TGTGTACCCACTCTTGGGTTACCTGGTTCGTGTGCAGCTCATGGGCCAAATCTTCGGAAAC CACTACCCTGGCTTCTTGCATGTCTTCGTTCTGAACGTGTTCGTTGTGGGCGCAGGAGTC CTGATGGCGAGGTTCTACCCCAACATCGGCTCCATCATTAGATACAGCGGTGCTCTCTG CGGCTTGGCCCTGGTGTTCGTCCTCCCATCCTTGATTCACATGGTGAGCTTGAAGAG GAGAGGAGAACTGCGTTGGACCTCCACTCTCTTCCATGGTTTCTTGATCCTGCTCGGCG TCGCTAACTTGCTGGGACAATTCTTCATGTAATAAGCTT	GenScript
drSLC38A9, H81W	CCATCACCACCACCATCACCATCACCTCGTGCCCAGGGGATCCATGGACGAGGATTCTAA GCCGCTGCTCGGATCAGTGCCTACCGGTGACTACTACACTGACTCCCTGGACCCTAAAC AAAGGAGGCCATTCCACGTGGAGCCAAGGAACATCGTCGGCGAGGATGTTCAAGAACGTG TGAGCGCTGAAGCTGCTGTGCTGTCCAGCAGGGTCCACTACTACTCTCGTCTCACCGGATC TTCAGACAGGTTGCTGGCACCTCCCGATCACGTGATTCCGTCTTGGGAAGACATCTACATTT ACTCACCACTGGGCACCGCATTCAAGGTCCAGGGTGGCGATAGTCCAATCAAAAACCCGTC GATCGTTACTATTTTCGCGATCTGGAACACCATGATGGGAACTAGTATTCTGTCGATCCCTTG GGGTATCAAGCAAGCTGGATTCACCCTCGGTATCATTATCATTGTGCTGATGGGACTCTTGA CTCTCTACTGCTGTTACCGCGTTCTGAAGTCCACAAAAAGCATCCCTTACGTGGACACGAG CGATTGGGAGTTCCCCGACGTCTGCAAGTACTACTTCGGAGGTTTCGGCAAATGGAGTTCG CTGGTCTTCTCTCTCGTTTCATTGATCGGAGCTATGGTGGTCTACTGGGTCCTCATGTCAAA CTTCTTGTTCAACACTGGCAAGTTCATTTTCAACTACGTTCACAACGTGCAGACGAGTGACGC TTTCGGTACACAGGGCACGGAGCGTGTTATCTGTCCTTACCCCGACGTGGACCCTCACGG CCAGTCCAGCACCAGTTTGTACTCCGGCAGCGACCAGTCGACTGGACTGGAATTCGATCAT TGGTGGTCTAAGACAAACACGATCCCCTTCTACCTGATTCTGCTCTTGCTGCCACTCTTGAAC TTCAGGTCTGCTTCATTCTTCGCCAGATTCACATTCCTGGGAACGATTTCAGTCATCTACCTG ATTTTCCTCGTTACTTACAAAGCCATCCAGCTGGGTTTCCACCTCGAGTTCCATTGGTTCGA CTCTTCAATGTTCTTCGTGCCCGAATTCAGGACATTGTTCCCACAGCTGTCCGGCGTCCTCA CGTTGGCTTTCTTCATCCACAACTGCATCATTACACTGATGAAGAACAACAAACATCAAGAGAA CAACGTTAGAGACCTGAGCCTCGCCTACCTGCTCGTGGGTTTGACCTACCTGTACGTCGGC GTTCTCATCTTCGCAGCGTTCCCAAGTCCACCGTTGTCGAAGGAGTGTATCGAACCGAACTT CCTCGACAACTTCCCTAGTTCGGATATTTTGGTGTTCGTCGCTCGCACATTCTTGCTGTTCC AGATGACCACTGTGTACCCACTCTTGGGTTACCTGGTTCGTGTGCAGCTCATGGGCCAAATC TTCGGAAACCACTACCCTGGCTTCTTGCATGTCTTCGTTCTGAACGTGTTCGTTGTGGGCGC AGGAGTCCTGATGGCGAGGTTCTACCCCAACATCGGCTCCATCATTAGATACAGCGGTGCT CTCTGCGGCTTGGCCCTGGTGTTCGTCCTCCCATCCTTGATTCACATGGTGAGCTTGAAG AGGAGAGGAGAACTGCGTTGGACCTCCACTCTCTTCCATGGTTTCTTGATCCTGCTCGGCG TCGCTAACTTGCTGGGACAATTCTTCATGTAATAAGCTT	GenScript
drSLC38A9, Y87F	CCATCACCACCACCATCACCATCACCTCGTGCCCAGGGGATCCATGGACGAGGATTCTAAGC CGCTGCTCGGATCAGTGCCTACCGGTGACTACTACACTGACTCCCTGGACCCTAAACAA AGGAGGCCATTCCACGTGGAGCCAAGGAACATCGTCGGCGAGGATGTTCAAGAACGTG TGAGCGCTGAAGCTGCTGTGCTGTCCAGCAGGGTCCACTACTACTCTCGTCTCACCGGATC TTCAGACAGGTTGCTGGCACCTCCCGATCACGTGATTCCGTCTCATGAAGACATCTACATTTTC TCACCACTGGGCACCGCATTCAAGGTCCAGGGTGGCGATAGTCCAATCAAAAACCCGTCGAT CGTTACTATTTTCGCGATCTGGAACACCATGATGGGAACTAGTATTCTGTCGATCCCTTGGGG TATCAAGCAAGCTGGATTCACCCTCGGTATCATTATCATTGTGCTGATGGGACTCTTGACTCTC TACTGCTGTTACCGCGTTCTGAAGTCCACAAAAAGCATCCCTTACGTGGACACGAGCGA TTGGGAGTTCCCCGACGTCTGCAAGTACTACTTCGGAGGTTTCGGCAAATGGAGTTCGCTG GTCTTCTCTCTCGTTTCATTGATCGGAGCTATGGTGGTCTACTGGGTCCTCATGTCAAACTTC TTGTTCAACACTGGCAAGTTCATTTTCAACTACGTTCACAACGTGCAGACGAGTGACGCTTT CGGTACACAGGGCACGGAGCGTGTTATCTGTCCTTACCCCGACGTGGACCCTCACGGCC AGTCCAGCACCAGTTTGTACTCCGGCAGCGACCAGTCGACTGGACTGGAATTCGATCATTG GTGGTCTAAGACAAACACGATCCCCTTCTACCTGATTCTGCTCTTGCTGCCACTCTTGAAC TTCAGGTCTGCTTCATTCTTCGCCAGATTCACATTCCTGGGAACGATTTCAGTCATCTACCT GATTTTCCTCGTTACTTACAAAGCCATCCAGCTGGGTTTCCACCTCGAGTTCCATTGGTTCG ACTCTTCAATGTTCTTCGTGCCCGAATTCAGGACATTGTTCCCACAGCTGTCCGGCGTCCTC ACGTTGGCTTTCTTCATCCACAACTGCATCATTACACTGATGAAGAACAACAAACATCAAGAGA ACAACGTTAGAGACCTGAGCCTCGCCTACCTGCTCGTGGGTTTGACCTACCTGTACGTCGG CGTTCTCATCTTCGCAGCGTTCCCAAGTCCACCGTTGTCGAAGGAGTGTATCGAACCGAAC TTCCTCGACAACTTCCCTAGTTCGGATATTTTGGTGTTCGTCGCTCGCACATTCTTGCTGTTC CAGATGACCACTGTGTACCCACTCTTGGGTTACCTGGTTCGTGTGCAGCTCATGGGCCAAA TCTTCGGAAACCACTACCCTGGCTTCTTGCATGTCTTCGTTCTGAACGTGTTCGTTGTGG GCGCAGGAGTCCTGATGGCGAGGTTCTACCCCAACATCGGCTCCATCATTAGATACAGCG GTGCTCTCTGCGGCTTGGCCCTGGTGTTCGTCCTCCCATCCTTGATTCACATGGTGAGC TTGAAGAGGAGAGGAGAACTGCGTTGGACCTCCACTCTCTTCCATGGTTTCTTGATCCTGC TCGGCGTCGCTAACTTGCTGGGACAATTCTTCATGTAATAAGCTT	GenScript
Recombinant DNA		
pFastBac1	Invitrogen Life TechnologiesBac-to-Bac^™^ Baculovirus Expression System	Cat#10359016
Software and Algorithms		
PHENIX	Adams et al. (2010)	https://www.phenix-online.org/

## Highlights

- A structure of SLC38A9 from *Danio rerio* (drSLC38A9) has been determined
- The N terminus (N-plug) of drSLC38A9 is folded and inserted in the transmembrane domain
- N-plug mediates amino acid transport of drSLC38A9
- Model of N-plug-mediated amino acid sensing by SLC38A9 in mTORC1 signaling is proposed
