# Supplementary material for: A Conformational Change in the N Terminus of SLC38A9 Signals mTORC1 Activation
Source: Structure. Author manuscript; Available in PMC 2023 Mar 8. (PMC9994763; doi:10.1016/j.str.2020.11.014)
Supplement: 1 [file NIHMS1878588-supplement-1.pdf]

Structure, Volume *29*

Supplemental Information

A Conformational Change in the N Terminus of SLC38A9 Signals mTORC1 Activation

Hsiang-Ting Lei, Xuelang Mu, Johan Hattne, and Tamir Gonen

Fig. S1

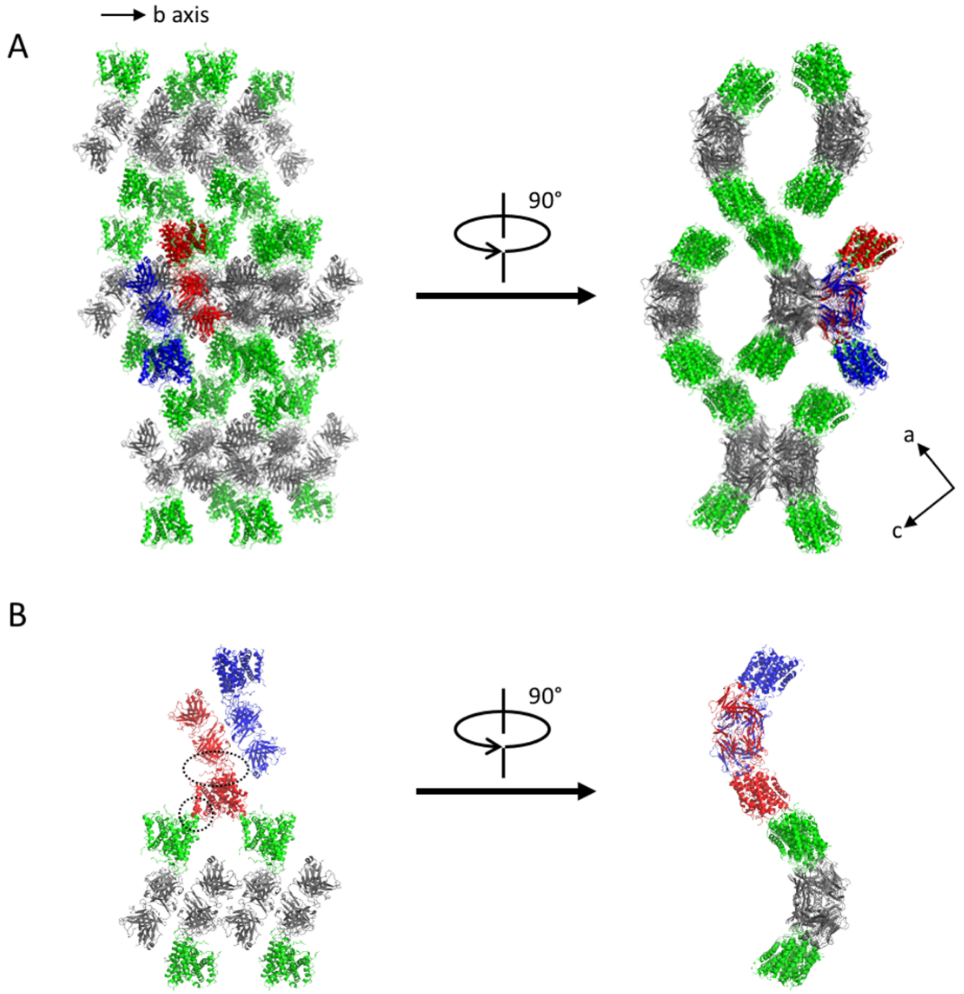

Fig. S1. Crystal packing and asymmetric unit. Related to Table 1 (**A**) Crystal packing showing SLC38A9-Fab complex lattice. Fab fragments (grey) form continuous layers in the crystallographic *b* axis, which are connected by SLC38A9 (green) layers along the crystallographic *ac* plane in a propeller-like head-to-head manner. One asymmetric unit is selected to show the structural block comprising two-fold SLC38A-Fab molecules (red and blue). (**B**) Interactions between SLC38A9 molecules and Fab fragments. One SLC38A9 (red) makes biological contacts with the complementary determining regions (CDRs) of a Fab (red) by its luminal loops. It also has interactions between Loop 8-9 (red) and Loop 10-11 (green), which appear to be crystal contacts and non-specific.

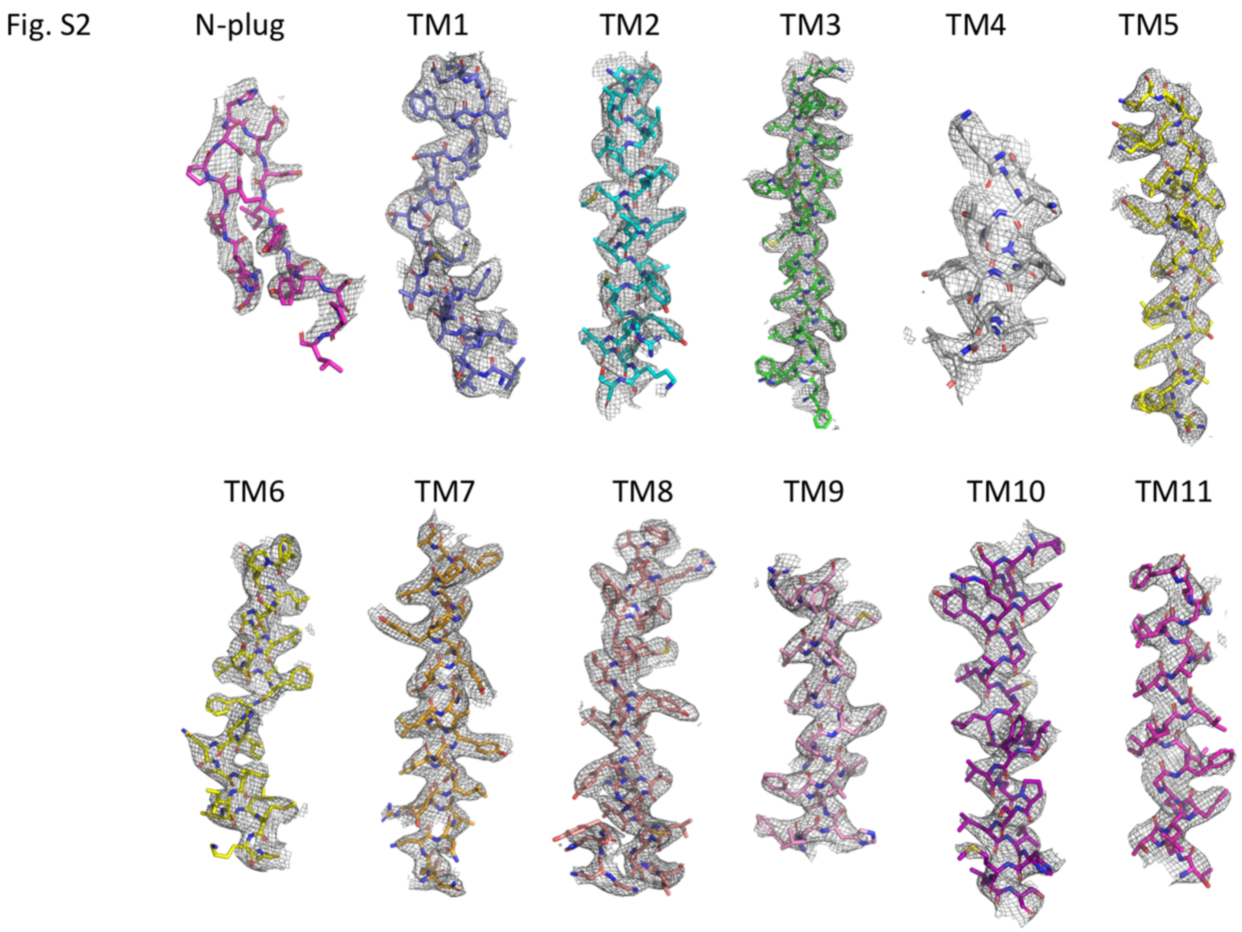

Fig. S2. Overall experimental density of N-plug and membrane helices of drSCL38A9 are shown with 2Fo-Fc map contoured at 1.2 σ (gray mesh). Related to Figure 1

Fig. S3

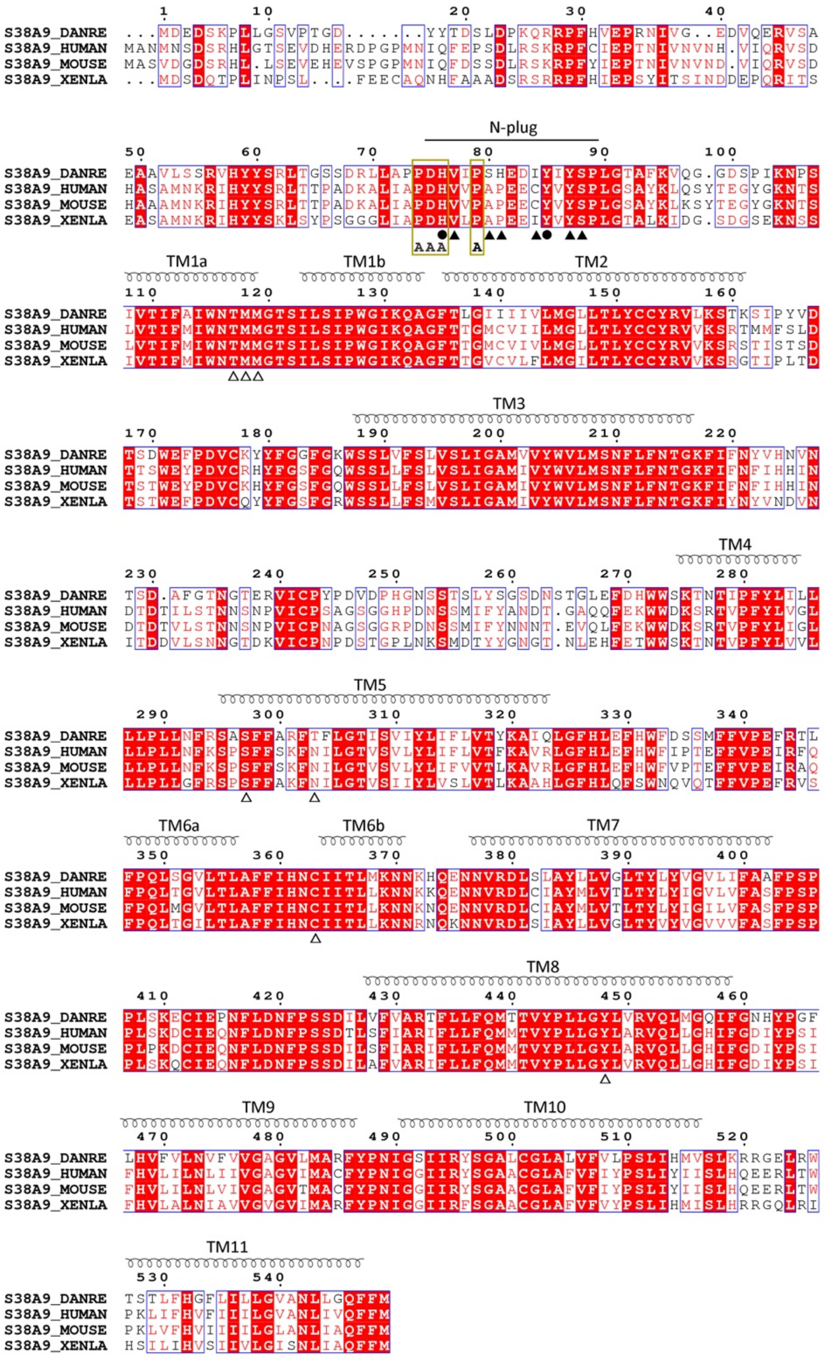

Fig. S3. Sequence alignment of SLC38A9 from zebrafish, human, mouse and clawed frog.

Related to Figure 2

The N-plug of drSLC38A9 (from Asp 75 to Pro 89) is noted. Residues which have hydrogen bonds between the N-plug and transmembrane helices were labeled by triangles, while residues forming intra-interactions of N-plug were marked with circles. Alanine substitutions of residues in yellow box will abolish the binding of hSLC38A9 to downstream Rag GTPase complex.

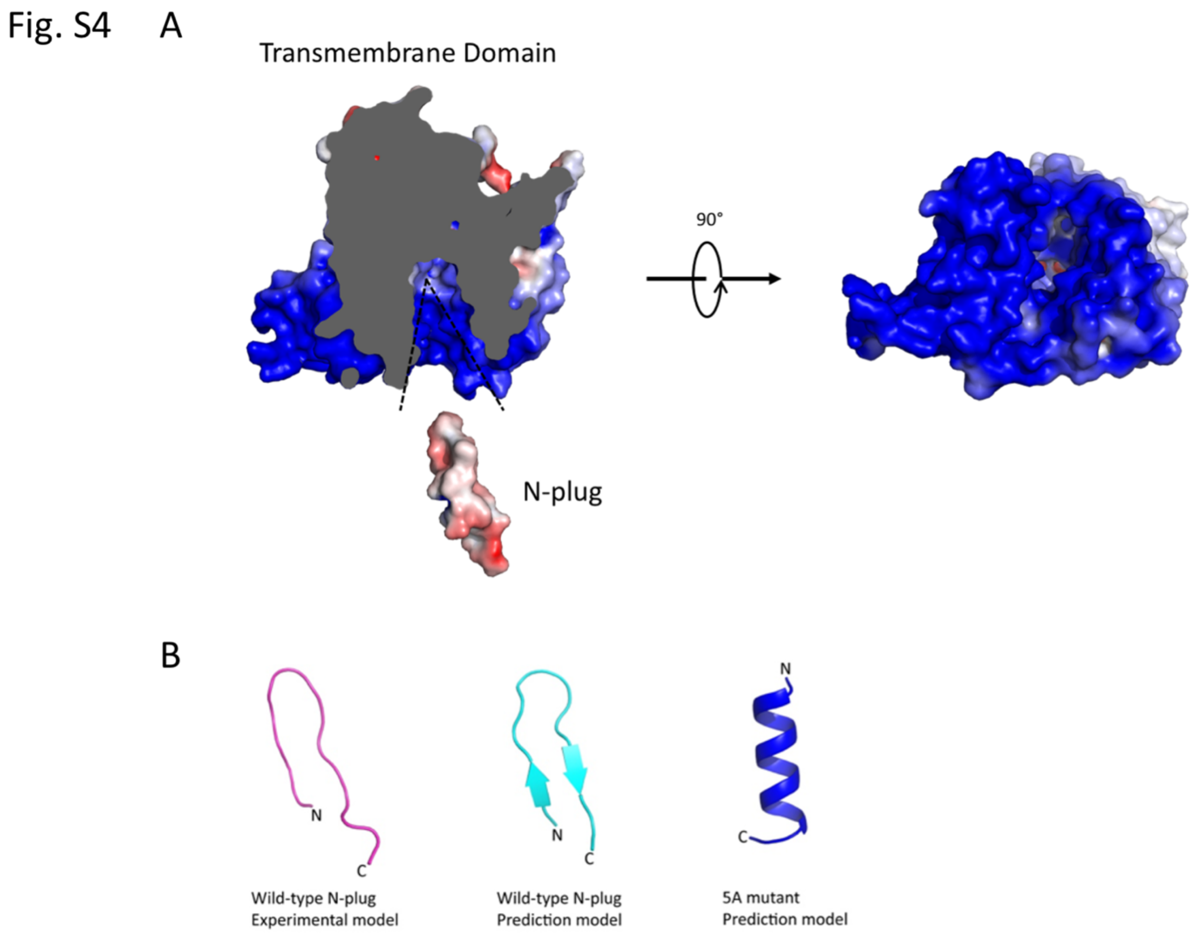

Fig. S4. Electrostatic surface representation and folding of N-plug for drSLC38A9.
Related to Figure 2
(A) N-plug has negatively charged surfaces and blocks the access leading to cytosolic side, which is dominantly positively charged. (B) The 5A mutations (P79A, S80A, H81A, E82A, and Y85A) of N-plug disrupt its secondary structure. P79A, S80A, H81A, and E82A locate in the beta-turn motif. Y85A breaks hydrogen bonds of the beta-sheet network (Shen *et al.*, 2014).

Fig. S5

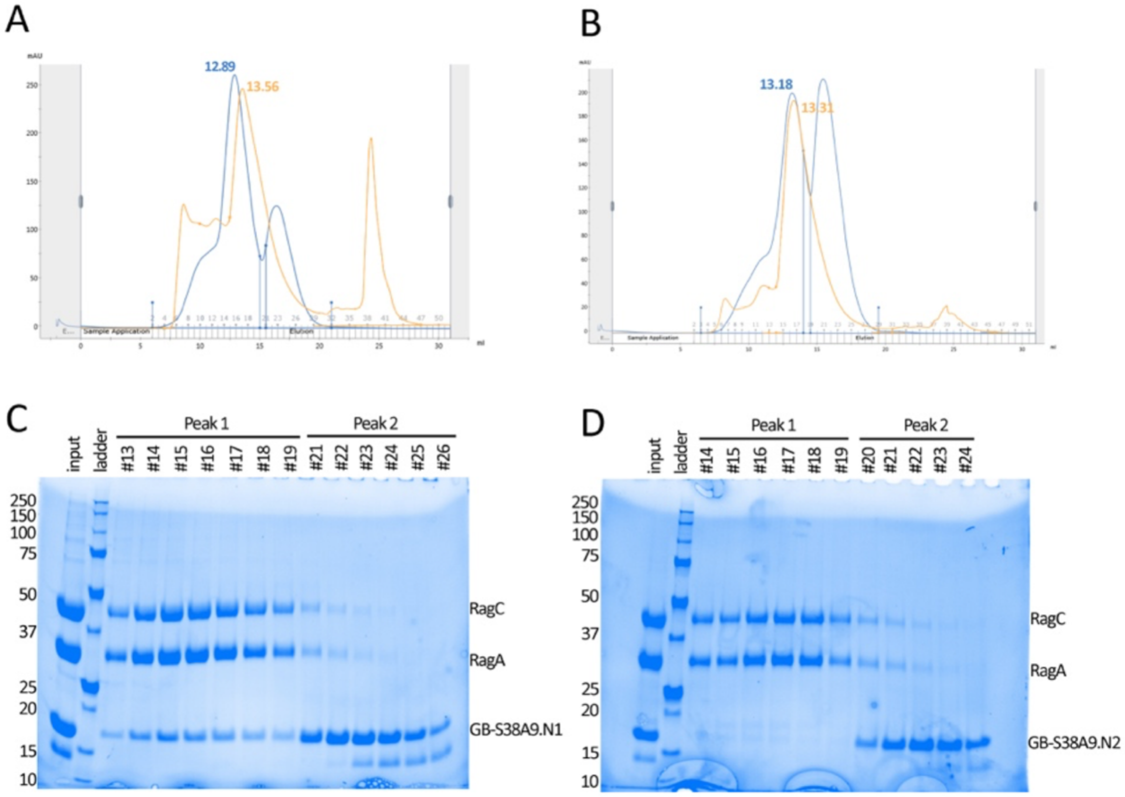

Fig. S5. Co-purification of GB1 domain tagged N-terminal fragments of drSLC38A9 with zebrafish Rag GTPase complex. Related to STAR Methods (A) The chromatogram displays a blue line with one peak at 12.89 mL retention volume (fractions 13-19) corresponding to drSLC38A9.N1 (1-96) and Rag GTPase complex and a second peak (fractions 21-26) corresponding to unbound N-terminal fragments. The orange superimposed curve depicts the zebrafish Rag GTPase complex eluted in the same column. Apparent peak shift was observed for the formation of drSLC38A9.N1 (1-96) and Rag GTPase complex. (B) Size exclusion chromatography profile of drSLC38A9.N1 (1-70) and Rag GTPase. No conspicuous peak shift was observed. (C and D) Fractions selected in (A) and (B) was sampled and analyzed on SDS-PAGE, including the input controls.
